# Efficacy of Motion-Sensing Game–Assisted Pulmonary Rehabilitation in Patients With Chronic Obstructive Pulmonary Disease: Systematic Review and Meta-Analysis of Randomized Controlled Trials

**DOI:** 10.2196/69562

**Published:** 2025-05-29

**Authors:** Cuirong Hu, Xia Lan, Yanwen Luo, Shu Zhu, Guilan Cheng

**Affiliations:** 1 Division of Internal Medicine, Institute of Integrated Traditional Chinese and Western Medicine, West China Hospital, Sichuan University/West China School of Nursing, Sichuan University, No.37 Guoxue Street, Wuhou District, Chengdu, Sichuan Province, 610041, China, 86 18980602084

**Keywords:** motion-sensing game, chronic obstructive pulmonary disease, pulmonary rehabilitation, exercise capacity, meta-analysis

## Abstract

**Background:**

While motion-sensing game (MSG) platforms provide immersive, real-time feedback environments for rehabilitation, research findings on their effectiveness in chronic obstructive pulmonary disease (COPD) pulmonary rehabilitation remain heterogeneous.

**Objective:**

This study aims to systematically evaluate the efficacy of MSG-assisted pulmonary rehabilitation for patients with COPD.

**Methods:**

This meta-analysis was conducted in accordance with the *Cochrane Handbook for Systematic Reviews of Interventions* and the PRISMA (Preferred Reporting Items for Systematic Reviews and Meta-Analyses) 2020 statement. Eight electronic databases (PubMed, Cochrane Library, Embase, CINAHL, Web of Science, Scopus, China National Knowledge Infrastructure, and Wanfang) were systematically searched from inception to March 2025. Randomized controlled trials (RCTs) comparing MSG-assisted versus conventional pulmonary rehabilitation in patients with COPD were included. Data analysis was performed using Review Manager 5.3 (Cochrane Collaboration) and Stata 17 (StataCorp LLC). Mean differences (MDs) and odds ratios (ORs) with 95% CIs were calculated. Risk of bias was assessed using the revised Cochrane Risk of Bias tool (Cochrane Collaboration), and evidence quality was evaluated using the Grading of Recommendations Assessment, Development, and Evaluations (GRADE) approach.

**Results:**

Analysis of 12 RCTs (n=776) demonstrated that MSG-assisted pulmonary rehabilitation, compared with conventional pulmonary rehabilitation, significantly improved 6-Minute Walk Distance (MD 23.23, 95% CI 14.47‐31.99; *P*<.001), upper limb strength via 30-Second Arm Curl Test (MD 1.83, 95% CI 0.63‐3.03; *P*=.003), balance performance (Balance Evaluation Systems Test; MD 2.34, 95% CI 1.52‐3.17; *P*<.001), and exercise adherence (OR 3.00, 95% CI 1.38‐6.52; *P*=.005). Additionally, significant improvements were observed in dyspnea severity (MD −0.25, 95% CI −0.48 to −0.02; *P*=.03), health-related quality of life (MD −6.00, 95% CI −10.96 to −1.04; *P*=.02), and psychological outcomes including anxiety (MD −2.41, 95% CI −3.42 to −1.39; *P*<.001) and depression (MD −1.40, 95% CI −2.69 to −0.42; *P*=.03). The overall methodological quality of the included studies was suboptimal with most evidence rated as “low” or “very low” quality.

**Conclusions:**

MSG-assisted pulmonary rehabilitation demonstrates significant improvements in exercise capacity, respiratory symptoms, quality of life, and psychological well-being among patients with COPD. Despite potential benefits, the predominance of low-quality evidence highlights the necessity for risk-benefit assessment before clinical implementation. Future research priorities should include larger, methodologically rigorous RCTs, standardized intervention protocols, investigation of sustained therapeutic effects, and cost-effectiveness analyses to establish definitive evidence for optimal implementation of gaming technology in pulmonary rehabilitation programs.

## Introduction

Chronic obstructive pulmonary disease (COPD) is characterized by persistent airflow limitation resulting from a complex interplay of airway inflammation, parenchymal destruction, and systemic manifestations [1]. This pathophysiological process extends beyond the lungs to induce skeletal muscle dysfunction, which is characterized by reduced muscle mass, strength, and endurance, and significantly contributes to exercise intolerance and impaired quality of life [1]. COPD represents a significant global health challenge, affecting over 400 million individuals worldwide and is projected to become the third leading cause of death by 2030 [2]. The economic burden of COPD continues to rise, with annual direct health care costs exceeding US $50 billion in high-income countries [3]. While pulmonary rehabilitation (PR), particularly exercise training, has been established as a cornerstone intervention that significantly enhances exercise capacity, alleviates symptoms, and improves quality of life [4,5], its implementation faces considerable challenges. Traditional programs struggle with issues of accessibility and adherence, reaching only 10%‐20% of eligible patients, primarily due to geographical barriers, limited health care resources, and lack of motivation [6,7].

The recent digital health revolution, accelerated by the COVID-19 pandemic, has catalyzed innovative approaches to health care delivery [7]. Motion-sensing game–assisted pulmonary rehabilitation (PR+MSG) has emerged as a promising solution, leveraging advanced technologies such as Nintendo Wii, Xbox Kinect, and Subor systems [8]. These platforms offer unique advantages, including real-time movement tracking and immediate feedback capabilities, providing superior accessibility and user-friendliness compared with conventional mobile apps, telehealth systems, and complex virtual reality setups [9,10]. The immersive and gamified nature of motion-sensing game (MSG)–assisted rehabilitation creates an engaging environment that effectively addresses both the physical and psychological aspects of rehabilitation, potentially overcoming the longstanding challenges of traditional rehabilitation programs by enhancing motivation and adherence [11].

While several randomized controlled trials (RCTs) have demonstrated the potential benefits of PR+MSG in the management of COPD [12], research results remain inconsistent [13]. Furthermore, previous systematic reviews and meta-analyses have examined technology-enhanced exercise interventions—including mobile apps, web-based platforms, telehealth systems, and virtual reality interfaces—in chronic respiratory conditions. However, they either encompassed a broader range of technologies or focused on specific outcomes [14,15]. As of the study period, no comprehensive meta-analysis has specifically evaluated the efficacy of PR+MSG across multiple outcomes in patients with COPD. Therefore, this meta-analysis aims to assess the effects of PR+MSG on exercise capacity, symptoms, quality of life, and psychological outcomes in patients with COPD. The findings will provide crucial evidence to inform the implementation of MSG in PR programs.

## Methods

### Search Strategy

We conducted a comprehensive search of PubMed, Cochrane Library, Embase, CINAHL, Web of Science, Scopus, China National Knowledge Infrastructure, and Wanfang databases from inception to March 2025, without language restrictions. Additionally, we manually searched the references of included studies and relevant published reports to identify eligible studies. A search strategy was developed for PubMed and the other databases (Table S1 in Multimedia Appendix 1).

### Inclusion and Exclusion Criteria

We selected the studies based on the following inclusion and exclusion criteria (Textbox 1).

Textbox 1. **Inclusion and exclusion criteria.**

**Inclusion criteria**

- Randomized controlled trials (RCTs) evaluating motion-sensing game (MSG)–assisted exercise training.
- Participants with chronic obstructive pulmonary disease (COPD) diagnosed according to the Global Initiative for Chronic Obstructive Lung Disease guidelines.
- Intervention group using MSGs from any MSG system (including Nintendo Wii, Subor, EyeToy, Kinect, etc) during pulmonary rehabilitation (PR) and control group receiving routine PR without game assistance.
- Measured outcomes including: (1) exercise capacity (6-Minute Walk Distance, 30-Second Arm Curl Test, Brief Balance Evaluation Systems Test, and completion rate of exercise training), (2) health-related quality of life (HRQL) measured using disease-specific or generic HRQL instruments, for example, COPD Assessment Test score, Chronic Respiratory Disease Questionnaire, and St. George’s Respiratory Questionnaire, (3) dyspnea severity (eg, the Medical Research Council scale and the modified Medical Research Council scale), and (4) psychological state (eg, Hospital Anxiety and Depression Scale, Self-Rating Depression Scale, and Self-Rating Anxiety Scale).

**Exclusion criteria**

- Participants with non-COPD respiratory diseases or other significant conditions.
- Abstract-only publications without full text or efficacy reporting.
- Studies with incomplete data or protocols only.

### Study Selection

The search was conducted by the first author and recorded in a Microsoft Excel file, from which duplicate entries were subsequently removed. Two independent reviewers (CH and XL) manually screened each title and abstract, and if necessary, a third reviewer (SZ) was consulted to resolve any disagreements. All full texts that met the inclusion criteria were included in the analysis.

### Data Extraction

Two researchers (CH and XL) independently extracted and recorded the following information from the enrolled studies: (1) study characteristics (author, publication year, country, study design, sample size, age, sex, and PR exercise training program), (2) methodological characteristics, and (3) outcome indicators (exercise capacity, health-related quality of life (HRQL), severity of dyspnea, and psychological state). Any disagreements were resolved by a third researcher (SZ).

### Quality Assessment

The risk of bias in individual studies was evaluated by two independent researchers (CH and XL) using the revised Cochrane Risk of Bias tool [16], which assesses the randomization process, adherence to interventions, completeness of outcome data, outcome measurement, and selection of reported results. The quality was rated as “low risk,” “some concerns,” or “high risk.” The Grading of Recommendations Assessment, Development, and Evaluations (GRADE) approach was employed to assess the quality of evidence for individual outcomes [17]. The quality of evidence was classified as high, moderate, low, or very low by evaluating five domains: risk of bias, inconsistency, indirectness, imprecision, and publication bias. Any disagreements were resolved by a third researcher (SZ).

### Statistical Analysis

Data analysis was conducted using Review Manager 5.3 (The Cochrane Collaboration) and Stata 17 (StataCorp LLC). To address potential baseline imbalances, we performed a pooled analysis using change scores, defined as the difference between baseline and postintervention values [18]. A random-effects model was used to estimate the aggregated outcomes. For dichotomous variables, we calculated odds ratios (ORs) with 95% CIs, while for continuous variables, mean differences (MDs) with 95% CIs were used. The overall results of the meta-analysis were visualized with forest plots. Forest plots visually summarize meta-analysis results by displaying individual study effect sizes (eg, risk ratios) with CIs, weighted summary effects, and heterogeneity metrics such as *I*². Heterogeneity among studies was assessed using Cochran Q test and quantified with the *I*² statistic. We interpreted *I*² values of 25%, 50%, and 75% as indicative of low, moderate, and high heterogeneity, respectively. Statistical significance was established at a 2-tailed *P* value of less than .05. The credibility of the evidence was evaluated using the GRADE approach, implemented through GRADEpro GDT software (Cochrane Collaboration). To assess potential publication bias, we generated funnel plots for outcomes with 10 or more studies and conducted Egger and Begg tests when appropriate. Subgroup analyses were performed based on the MSG system used (Xbox Kinect, Nintendo Wii, or Subor) to explore potential sources of heterogeneity and evaluate the consistency of treatment effects across different platforms.

### Ethical Considerations

This systematic review and meta-analysis was conducted in accordance with the *Cochrane Handbook for Systematic Reviews of Interventions* and the PRISMA (Preferred Reporting Items for Systematic Reviews and Meta-Analyses) 2020 statement (Checklist 1). The protocol was registered with the International Prospective Register of Systematic Reviews (PROSPERO; CRD42024603044). Since the data for this study were derived from previously published research, ethical approval and informed consent were not required.

## Results

### Flow and Characteristics of Included Studies

**Figure 1.**
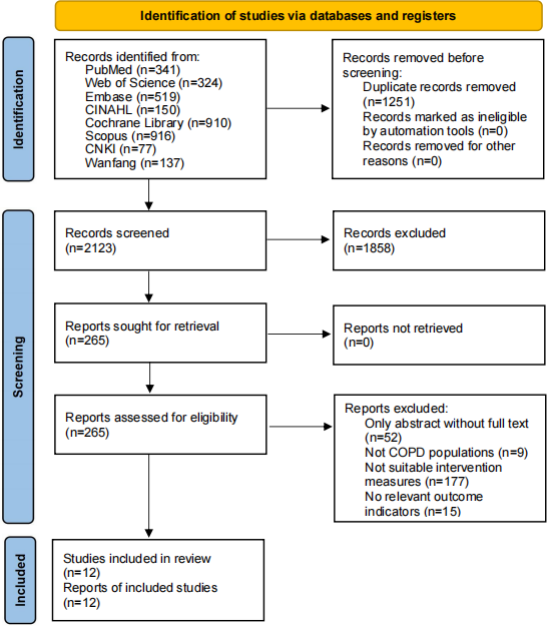
Flow diagram of the study selection procedure for the meta-analysis of randomized controlled trials investigating the efficacy of motion-sensing game–assisted pulmonary rehabilitation compared with routine pulmonary rehabilitation in patients with chronic obstructive pulmonary disease, following Preferred Reporting Items for Systematic reviews and Meta-Analyses (PRISMA) guidelines. CNKI: China National Knowledge Infrastructure; COPD: chronic obstructive pulmonary disease.

The flow diagram of the selection procedure is presented in Figure 1. Initially, 3374 papers were identified through database searches. After the removal of duplicates, 2123 papers remained for the first screening. Following a review of titles and abstracts, 265 papers were selected for full-text evaluation, resulting in the exclusion of 1858 papers. Ultimately, 12 trials involving 776 participants met the inclusion criteria and were incorporated into the quantitative synthesis (Figure 1). Tables1  2 summarize the characteristics of the included studies, which were published between 2014 and 2025. The majority of these studies were conducted in China (n=8) [12,19-25], while the remainder were distributed across Poland (n=2) [13,26], Indonesia (n=1) [27], and Italy (n=1) [28]. Intervention durations ranging from 2 to 18 weeks, most commonly between 6 and 8 weeks. A total of 3 main gaming systems were used: the Subor systems (6 studies) [12,19-23], Kinect-based systems (4 studies) [13,24-26], and Nintendo Wii systems (2 studies) [27,28]. Sessions typically lasted 20 to 30 minutes and were conducted 5 times weekly under professional supervision with established safety monitoring protocols.

**Table 1.** Characteristics of randomized controlled trials evaluating motion-sensing game–assisted pulmonary rehabilitation (PR+MSG) versus routine pulmonary rehabilitation in patients with chronic obstructive pulmonary disease. Data are presented as absolute values and means (SD).^a^

First author (publication year)	Country	Sample size, n	Experimental group: PR+MSG		Control group: routine pulmonary rehabilitation		Duration of intervention	Reported outcomes
			Sample size, n	Age (years), mean (SD)	Sample size, n	Age (years), mean (SD)		
Jin et al (2025) [12]	China	74	36	73.97 (8.69)	38	74.00 (10.01)	6 wks	①③
Rutkowski et al (2020) [13]	Poland	72	38	60.6 (4.3)	34	62.1 (2.9)	2 wks	①②
Rutkowski et al (2019) [26]	Poland	68	34	60.5 (4.3)	34	62.1 (2.9)	2 wks	①②
Sutanto et al 2019 [27]	Indonesia	20	10	65.1 (7.5)	10	65.6 (4.7)	6 wks	①⑤
Mazzoleni et al (2014) [Bibr R28][28]	Italy	39	19	68.9 (11)	20	73.5 (9.2)	2-3 wks	①②⑤
Wei et al (2024)[Bibr R23] [23]	China	122	61	72.32 (6.38)	61	72.32 (6.38)	8 wks	①③⑤
Zhu et al (2021) [Bibr R25][25]	China	43	22	65.16 (12.78)	21	64.54 (12.89)	16 wks	①④⑤⑥
Zhou et al (2021) [Bibr R24][24]	China	119	61	70.54 (6.89)	58	71.38 (5.78)	18 wks	①④⑤
Jin (2021) [Bibr R19][19]	China	69	35	73.97 (8.69)	34	74.00 (10.01)	6 wks	①②⑦
Geng (2020) [21]	China	40	21	68.66 (6.41)	19	68.52 (5.76)	8 wks	①⑤⑥
Geng et al (2020) [22]	China	44	22	68.66 (6.41)	22	68.52 (5.76)	8 wks	⑦
Jin et al (2020) [Bibr R20][20]	China	66	33	74.15 (8.75)	33	73.97 (10.14)	Begin within 48 h of admission and continue until the day of discharge	③

a ① 6-Minute Walk Distance; ② 30-Second Arm Curl Test; ③ Brief Balance Evaluation Systems Test; ④ completion rate of exercise training; ⑤ severity of dyspnea; ⑥ quality of life; and ⑦ psychological state.

**Table 2.** Detailed characteristics of the experimental group evaluating PR+MSG^a^ in patients with chronic obstructive pulmonary disease.

First author (publication year)	PR+MSG	MSG^b^ system	Game	Implementation
Jin et al (2025)[Bibr R12] [12]	Patients received a once-daily, 20-min MSG session based on the control group.	The Little King somatosensory gaming system (Subor) consisted of a host console, motion sensors, and a display screen. The system features dual sensor technology for enhanced motion capture accuracy and real-time feedback.	Three games (“Kitchen Sharp Knife,” “Swimming Master,” and “Table Tennis Master”) were selected, and one-to-one instruction was implemented to ensure that the patients mastered the exercises correctly.	The exercise begins with a 3-min “Kitchen Sharp Knife” warm-up, continues for 5 min with the left hand only, 5 min with the right hand only, takes a 3-mins break, and concludes with a 4-min “Swimming Master” exercise. Patients can decide to sit or stand during exercise based on their comfort and overall health. Oxygen was administered if the patient’s oxygen saturation fell below 88%, and exercise resumed after stabilizing oxygen saturation. The exercise was discontinued if the Borg score exceeded 6 or adverse events occurred.
Rutkowski et al (2020) [13]	Patients participated in traditional pulmonary rehabilitation, complemented with endurance exercise training and Kinect system–based virtual reality sessions.	The system utilized an Xbox 360 console, a Kinect motion sensor, and a projector with speakers. Session intensity was controlled through Kinect Adventures games (Microsoft Game Studios, Washington, US).	Four Kinect games were selected and implemented: “20,000 Leaks,” “Curvy Creek,” “Rally Ball,” and “Reflex Ridge.”	Each patient participated in the 4 games in a fixed sequence during a 20-min session, conducted 5 d weekly with consistent workloads. Heart rate monitoring ensured patients remained below their age-predicted maximal heart rate (208‐0.7×age). A physiotherapist supervised all virtual reality training sessions. Exercise was modified or discontinued if patients showed signs of excessive exertion or if adverse events occurred.
Rutkowski et al (2019) [Bibr R26][26]	Patients received standard pulmonary rehabilitation plus once-daily Kinect system–based exercise sessions.	The system consisted of an Xbox 360 console, Kinect motion sensor, and a projector with speakers. The console was positioned one meter high on a table, with the Kinect sensor mounted on the projector, projecting images onto a wall 2.5 meters away. The designated play area measured 1.8×1.8 meters and was situated 1.2 meters from the Kinect sensor.	Four Kinect Adventures games were implemented: rafting, cross-country running, ball hitting, and roller-coaster ride.	Patients performed specific movements in front of the motion sensor following Kinect Adventures minigame protocols. Each game session began with the manufacturer’s instructions displaying game objectives and avatar control guidance.
Sutanto et al (2019) [Bibr R27][27]	Patients received standard hospital–based outpatient exercise training plus individualized 30-min Wii Fit gaming sessions in a dedicated room equipped with Nintendo Wii, balance board, and flat-screen TV.	The Nintendo Wii Fit gaming system included a Wii console, Wii balance board, and display screen.	Three specific Wii Fit exercises were implemented: yoga exercises (“Deep Breathing” and “Half Moon”), “Torso Twist,” and aerobic exercise (“Free Run”).	Each patient received initial one-to-one instruction for proper game execution. Sessions were performed standing on the Wii balance board (except during “Free Run”). Game duration, difficulty levels, and scores were documented. Exercise was discontinued if the pulse rate exceeded the Karnoven formula maximum, the respiratory rate exceeded 30/min, or SpO2 fell below 90%. All adverse events were recorded.
Mazzoleni et al (2014) [Bibr R28][28]	Patients participated in traditional pulmonary rehabilitation complemented by seven daily 1-h Wii Fit Plus exercise sessions during the final wk of a 3-wk rehabilitation program. Sessions were conducted on an individual basis in a dedicated room equipped with a TV screen.	The Nintendo Wii Fit Plus system included a Wii console, Wii Balance Board as a haptic sensor–based interface, and display. The patient’s movements were translated to an “avatar” in the games, providing visual and auditory feedback.	Three Wii Fit Plus activities were selected and implemented in sequence: (1) “Yoga” activity involving deep breathing in standing position on the balance board (5 min at start and end), (2) “Jogging Plus” with 10-min feedback-aided running on spot, (3) “Twisting and Squat” comprising 10-min feedback-aided trunk twisting and arm/leg squatting exercises.	Each session followed a standardized protocol with continuous monitoring of ECG, pulse oximetry, and respiratory rate. Exercise intensity was maintained to elicit dyspnea levels of 4‐6 on the modified Borg scale. Heart rate was kept below 80% of predicted maximum and oxygen saturation above 85%. Sessions were individually supervised by a physiotherapist. Exercise was modified or discontinued if heart rate exceeded limits, oxygen saturation fell below threshold, or if adverse events occurred.
Wei et al (2024) [Bibr R23][23]	Patients received routine pulmonary rehabilitation combined with MSG sessions during hospitalization. Training was conducted individually in a dedicated room based on the patient’s condition in a semirecumbent, sitting, or standing position.	The Little King somatosensory gaming system (Subor) consisted of a host console, motion sensors, and a display screen. The system features dual sensor technology for enhanced motion capture accuracy and real-time feedback.	Around 3 specific games were selected: (1) “Kitchen Sharp Knife” for a 3-min warm-up, (2) “Table Tennis Master” for a 10-min exercise (5 min each for left and right hands), and (3) “Swimming Master” for a 4-min exercise.	Each session lasted approximately 20 min with continuous monitoring of oxygen saturation, breathing, blood pressure, and heart rate. Patients could rest for 1 min if experiencing fatigue or dyspnea and resume after symptoms resolved. Oxygen was administered if saturation was low; exercise resumed after stabilization. The physiatrist provided initial one-on-one instruction to ensure proper game execution. For home-based training after discharge, patients maintained exercise logs with twice-weekly telephone follow-up for supervision and guidance during the 8-wk intervention period.
Zhu et al (2021) [Bibr R25][25]	Patients received standard rehabilitation combined with Kinect 2.0–based somatosensory game training. Training sessions were conducted under the supervision of nurses and rehabilitation physicians with initial demonstration and guidance.	The system utilized an XBOX360 console with a Kinect 2.0 motion sensor connected to an E9i projector for display. The setup provided real-time motion tracking and visual feedback.	Around 3 specific Kinect games were implemented in sequence: (1) “Cross-country Running” engaging in cardio exercise, (2) “Fruit Ninja” involving upper limb coordination, and (3) “Obstacle Skiing” combining balance and full-body movements. Each game was demonstrated with a detailed movement breakdown before patient participation.	Exercise sessions were conducted once daily, 5 times per wk. Session duration was progressively increased from an initial 15 min to 35 min per d (adding 10 min weekly). Health care professionals provided real-time correction of improper movements during training. The three games were performed in a fixed sequence under professional supervision to ensure proper form and safety.
Zhou et al (2021) [Bibr R24][24]	Patients received standard rehabilitation plus Kinect-based somatosensory game training. Training was conducted under the close supervision of rehabilitation physicians with vital signs monitoring through wearable pulse oximeters.	The system consisted of a control console, Kinect motion sensor, and a projector with speakers. The setup required specific positioning: console height at 1 meter, designated play area of 1.8×1.8 meters, positioned 1.2 meters from the Kinect sensor. Equipment calibration was performed before each session to ensure accurate motion tracking.	Around 4 Kinect games were implemented in sequence: (1) “Cross-country Running” for cardio exercise, (2) “Rafting” for balance training, (3) “Ball Hitting” for upper limb coordination, and (4) “Mountain Biking” for full-body movements. Each game sequence was preceded by professional demonstration and instruction.	Sessions lasted approximately 30 min, conducted once daily, 5 times per wk for 24 wks. A 1-min rest interval was provided between games. Training was performed under the arp Knifsupervision of health care professionals or family members with continuous monitoring through pulse oximetry connected to a home health management system. Patient vital signs were monitored in real-time, allowing physicians to review health status and adjust rehabilitation plans accordingly. Regular follow-up was maintained throughout the intervention period.
Jin (2021) [Bibr R19][19]	Patients received standard care plus early rehabilitation with somatosensory game training based on individualized exercise prescriptions. Training sessions were conducted under one-on-one professional guidance starting within 48 h of admission.	The Little King somatosensory gaming system (Subor) consisted of a host console, motion sensors, and a display screen. The system features dual sensor technology for enhanced motion capture accuracy and real-time feedback.	About 3 specific games were implemented in sequence: (1) “Kitchen Sharp Knife” for 3-min warm-up, (2) “Table Tennis Master” for bilateral upper limb training (5 min each hand), and (3) “Swimming Master” for 4-min full-body exercise. Games were selected to target different aspects of rehabilitation.	Sessions lasted 20 min total, conducted once daily, 5 d per wk for 6 wks. Exercise position could be adjusted between standing, sitting, or semirecumbent based on patient condition. Brief rest periods (15‐30 s) were allowed during games if patients experienced dyspnea or fatigue. Exercise intensity was maintained at Borg scale 3‐6. Vital signs (heart rate, blood pressure, respiratory rate, and oxygen saturation) and Borg scores were monitored before and after exercise. Oxygen was administered if saturation fell below 88%, with exercise resuming after stabilization. Exercise was discontinued if the Borg score exceeded 6 or adverse events occurred.
Geng (2020) [21]	Patients received standard guidance plus home-based upper limb training using somatosensory games. Initial instruction included game knowledge and operation methods with hands-on demonstration.	The Little King somatosensory gaming system (Subor) consisted of a host console, motion sensors, and a display screen. The system features dual sensor technology for enhanced motion capture accuracy and real-time feedback.	Three specific games were selected: (1) “Kitchen Sharp Knife,” (2) “Table Tennis Master,” and (3) “Boxing.” Each game targeted different aspects of upper limb rehabilitation, with detailed operation guides provided to patients.	After baseline assessment, researchers installed gaming equipment in patients’ homes. One-on-one instruction was provided until patients successfully demonstrated game mastery. Written operation manuals were distributed for reference. The 8-wk program consisted of 30-min sessions, 5 d per wk. The initial session was supervised for at least 30 min with SpO₂ monitoring and assessment of fatigue and dyspnea postexercise to ensure safety.
Geng et al (2020) [22]	Patients received standard health guidance plus home-based somatosensory game training. Training methods were individually instructed with regular follow-up through WeChat, phone calls, and home visits.	The Little King somatosensory gaming system (Subor) consisted of a host console, motion sensors, and a display screen. The system features dual sensor technology for enhanced motion capture accuracy and real-time feedback.	Three specific games were implemented: “Kitchen Sharp Knife” for warm-up and cool-down (3 min each, divided into 2×90 s sessions), followed by “Table Tennis Master” (15 min in 3×5 min sessions) and “Boxing” (9 min in 6×90 s sessions) as the main exercise components.	The 8-wk program consisted of daily 30-min sessions, conducted 5 d per wk. Exercise could be performed standing or seated, with brief rest periods (15‐30 s) allowed during games if experiencing dyspnea or fatigue. Exercise intensity was self-regulated using the modified Borg scale (maintaining 3‐5), with instructions to stop if experiencing shortness of breath, chest tightness, palpitations, or fatigue. Patients maintained exercise diaries documenting training date, duration, game types, intensity (Borg score), and subjective symptoms. Health care professionals regularly reviewed these diaries and provided individualized guidance through multiple communication channels.
Jin et al (2020) [Bibr R20][20]	Patients received standard care plus somatosensory game training guided by the early rehabilitation team. Training sessions were conducted under one-on-one professional instruction to ensure proper exercise technique.	The Little King somatosensory gaming system (Subor) consisted of a host console, motion sensors, and a display screen. The system features dual sensor technology for enhanced motion capture accuracy and real-time feedback.	Around 3 games were implemented in sequence: “Kitchen Sharp Knife” for warm-up (3 min), “Table Tennis Master” for bilateral upper limb training (5 min each hand), and “Swimming Master” for full-body exercise (4 min), with a 3-min rest period between activities.	Daily 20-min sessions could be performed in standing, sitting, or semirecumbent positions. Brief rest periods (15‐30 s) were allowed during games if experiencing dyspnea or fatigue. Exercise intensity was maintained at modified Borg scale 3‐6. Vital signs (heart rate, blood pressure, respiratory rate, and oxygen saturation) and the Borg scores were monitored before and after exercise. Oxygen was administered per physician orders if saturation fell below 88%, with exercise resuming after stabilization. Exercise was discontinued if Borg score exceeded 6 or adverse events occurred. The rehabilitation team performed daily assessments during hospitalization, documenting pre-exercise evaluation, exercise components, completion status, and postexercise assessment.

a PR+MSG: motion-sensing game–assisted pulmonary rehabilitation.

b MSG: motion-sensing game.

### Risk of Bias in the 12 Included Studies

Based on the revised Cochrane Risk of Bias tool assessment of these 12 RCTs, all studies demonstrated “Some concerns” in the overall risk of bias evaluation, with none rated as high risk. In specific domains, the randomization process exhibited mixed quality, with 33.3% (4/12) of studies rated as low risk and 66.7% (8/12) as having some concerns. All studies (12/12, 100%) were rated as “Some concerns” for deviations from intended interventions, indicating potential issues with protocol adherence. However, all studies demonstrated a low risk of bias regarding missing outcome data and the selection of reported results, reflecting good follow-up rates and appropriate outcome reporting. The measurement of outcomes was generally appropriate, with 66.7% (8/12) of studies showing low risk and 33.3% (4/12) showing some concerns. Further details are provided in Figure S1A and S1B in Multimedia Appendix 2.

### Outcomes

#### Exercise Capacity and Program Adherence

**Figure 2.**
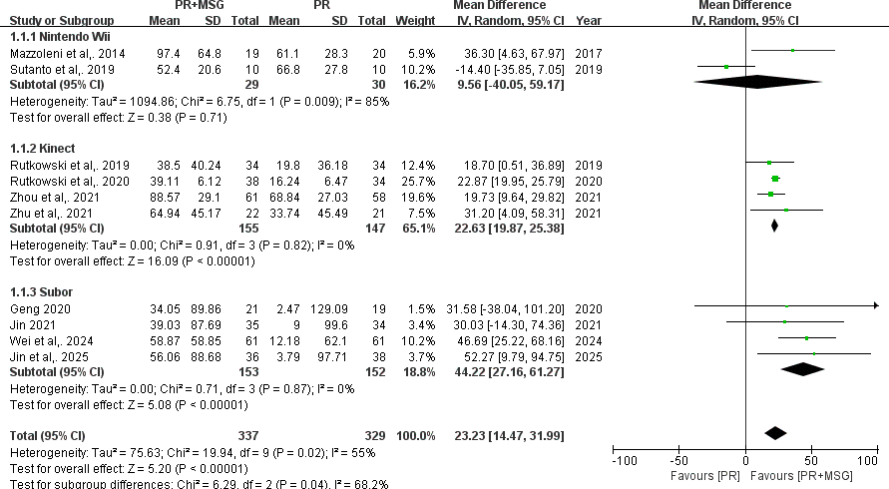
Forest plots depicting the effect of motion-sensing game–assisted pulmonary rehabilitation (PR+MSG) versus routine pulmonary rehabilitation (PR) on 6-Minute Walk Distance in patients with chronic obstructive pulmonary disease [12,13,19,21,23-28].

For the 6-Minute Walk Distance (6MWD), analysis of 10 RCTs (n=666) demonstrated that PR+MSG significantly improved walking distance compared with PR alone (MD 23.23, 95% CI 14.47-31.99 m; *P*<.001), with moderate heterogeneity (*I*²=55%). Subgroup analyses revealed that while Nintendo Wii–based interventions showed no significant advantage (MD 9.56, 95% CI −14.05 to 59.17 m; *P*=.71), both Kinect-based (MD 22.63, 95% CI 19.87-25.38 m; *P*<.001) and Subor-based systems (MD 44.22, 95% CI 27.16 to 61.27 m; *P*<.001) demonstrated significant improvements. Both Kinect and Subor subgroups display no heterogeneity (*I*²=0%) (Figure 2).

**Figure 3.**
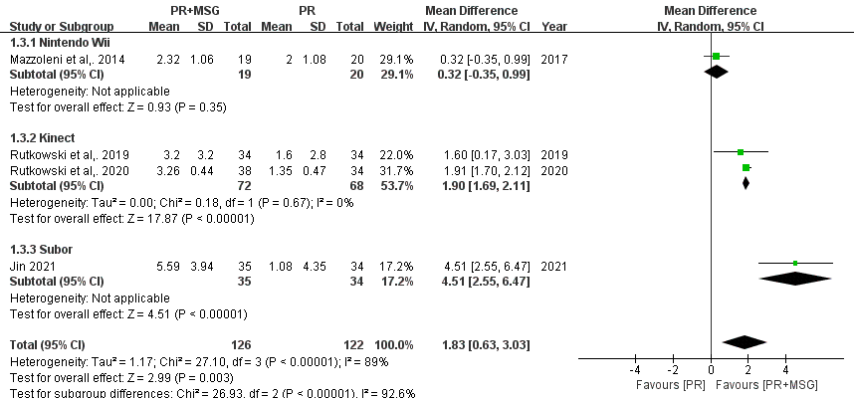
Forest plots depicting the effect of motion-sensing game–assisted pulmonary rehabilitation (PR+MSG) versus routine pulmonary rehabilitation (PR) on upper limb strength as measured by the 30-Second Arm Curl Test in patients with chronic obstructive pulmonary disease [13,19,26,28].

Analysis of the 30-Second Arm Curl Test from 4 RCTs (n=248) indicated that PR+MSG significantly enhanced upper limb muscle strength (MD 1.83, 95% CI 0.63-3.03 repetitions; *P*=.003), despite considerable heterogeneity (*I*²=89%). In subgroup analyses, Nintendo Wii–based training showed no significant improvement (MD 0.32, 95% CI −0.35 to 0.99; *P*=.35), while both Kinect-based (MD 1.90, 95% CI 1.69 to 2.11; *P*<.001) and Subor-based systems (MD 4.51, 95% CI 2.55-6.47; *P*<.001) demonstrated significant benefits (Figure 3).

**Figure 4.**
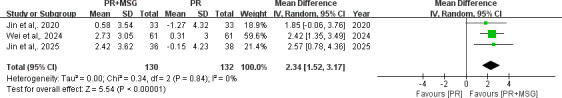
Forest plots depicting the effect of motion-sensing game–assisted pulmonary rehabilitation (PR+MSG) versus routine pulmonary rehabilitation (PR) on balance function assessed by the Brief Balance Evaluation Systems Test in patients with chronic obstructive pulmonary disease [12,20,23].

**Figure 5.**
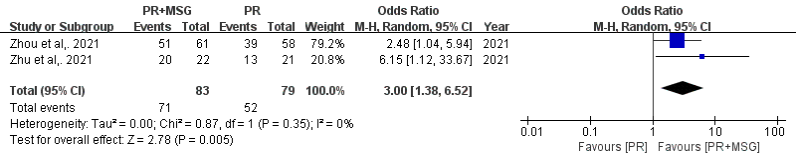
Forest plots illustrating the comparison of completion rates of exercise training between motion-sensing game–assisted pulmonary rehabilitation and routine pulmonary rehabilitation in patients with chronic obstructive pulmonary disease [24,25].

For balance function, assessed by the Brief Balance Evaluation Systems Test, analysis of 3 studies (n=262) revealed significant improvements with PR+MSG (MD 2.34, 95% CI 1.52-3.17 points; *P*<.001), with remarkable consistency across studies (*I*²=0%; *P*=.84) (Figure 4). Additionally, examination of exercise training completion rates from 2 studies (n=162) showed that PR+MSG significantly increased adherence (OR 3.00, 95% CI 1.38-6.52; *P*=.005), with no heterogeneity (*I*²=0%; *P*=.35). (Figure 5).

#### Severity of Dyspnea and Quality of Life

**Figure 6.**
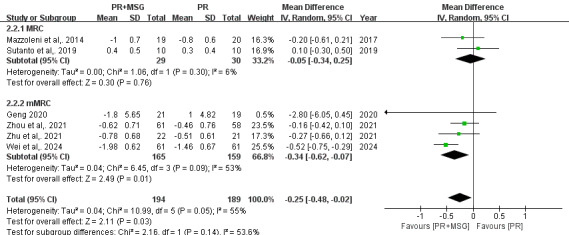
Forest plots depicting the effect of motion-sensing game–assisted pulmonary rehabilitation (PR+MSG) versus routine pulmonary rehabilitation (PR) on the severity of dyspnea in patients with chronic obstructive pulmonary disease [21,23-25,27,28].

Analysis of dyspnea severity encompassing 6 studies (n=383) demonstrated that PR+MSG significantly alleviated dyspnea compared with routine PR alone (MD −0.25, 95% CI −0.48 to −0.02; *P*=.03), with moderate heterogeneity (*I*²=55%). Subgroup analyses revealed differential effects based on assessment tools, while studies using the Medical Research Council (MRC) scale showed no significant improvement (MD −0.05, 95% CI −0.34 to 0.25; *P*=.76; *I*²=6%, 2 studies), those using the modified MRC scale demonstrated significant benefits (MD −0.34, 95% CI −0.62 to −0.07; *P*=.01; *I*²=53%, 4 studies) (Figure 6).

**Figure 7.**
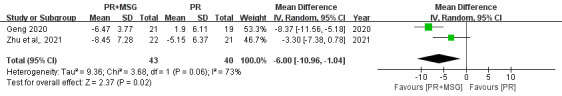
Forest plots depicting the effect of motion-sensing game–assisted pulmonary rehabilitation (PR+MSG) versus routine pulmonary rehabilitation (PR) on health-related quality of life in patients with chronic obstructive pulmonary disease [21,25].

For HRQL, analysis of 2 studies (n=83) using the COPD Assessment Test (CAT) showed significant improvements with PR+MSG (MD −6.00, 95% CI −10.96 to −1.04; *P*=.02). Despite moderate heterogeneity (*I*²=73%), both studies consistently indicated benefits favoring PR+MSG intervention (Figure 7).

#### Psychological State

**Figure 8.**
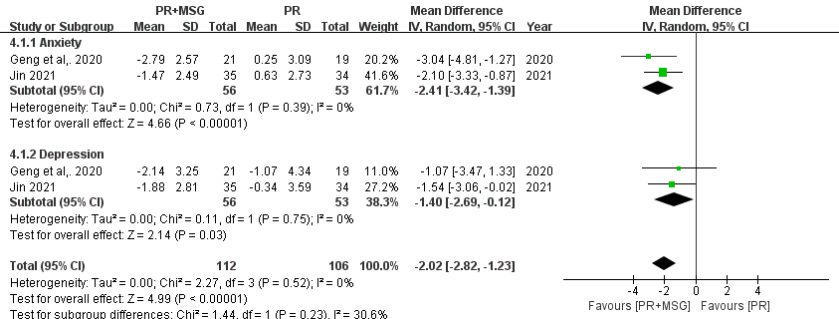
Forest plots depicting the effect of motion-sensing game–assisted pulmonary rehabilitation (PR+MSG) versus routine pulmonary rehabilitation (PR) on psychological outcomes in patients with chronic obstructive pulmonary disease [19,22].

Assessment of psychological outcomes using the Hospital Anxiety and Depression Scale from 2 studies (n=218) revealed significant improvements with PR+MSG for both anxiety (MD −2.41, 95% CI −3.42 to −1.39; *P*<.001) and depression (MD −1.40, 95% CI −2.69 to −0.42; *P*=.03). Notably, both psychological domains showed consistent benefits across studies with no heterogeneity (*I*²=0%), with anxiety demonstrating a more pronounced effect size compared with depression (Figure 8).

### Risk of Bias Within Outcomes

The quality of all synthesized evidence was assessed as “low” or “very low” using the GRADE approach (Table S2 in Multimedia Appendix 3). Sensitivity analysis used the selective model analysis method, wherein both fixed-effect and random-effect models were applied to the aforementioned experimental data, yielding consistent results. A leave-one-out analysis was conducted by sequentially excluding each study and re-estimating the analysis. This process revealed minimal changes in all indicators compared with the original analysis, suggesting low sensitivity and enhancing the robustness and reliability of the results. Additionally, the funnel plot for the 6MWD (10 studies, *P_Egger_*=0.69) showed no apparent publication bias.

## Discussion

### Principal Findings

This meta-analysis demonstrates that PR+MSG shows advantages over routine PR in improving exercise capacity, balance function, symptom control, quality of life, and psychological outcomes across multiple health domains. These findings provide scientific evidence supporting the integration of gaming technology into rehabilitation programs.

#### Exercise Capacity and Program Adherence

Our analysis demonstrates that PR+MSG significantly enhances exercise capacity across multiple domains, consistently outperforming conventional rehabilitation programs. Of particular note, newer systems such as Kinect and Subor show superior outcomes, likely attributable to their advanced motion capture capabilities and comprehensive whole-body engagement features. These systems effectively address multiple aspects of physical function simultaneously, including cardiorespiratory endurance, upper limb strength, and balance function [29].

The 6MWD, a critical functional parameter in patients with COPD, reflects overall physical endurance and directly correlates with disease severity, mortality risk, and performance in activities of daily living [30]. Similarly, improvements in upper limb strength assessed by the 30-Second Arm Curl Test address a frequently overlooked aspect of COPD-related dysfunction. Patients with COPD commonly experience upper limb muscle weakness resulting from respiratory muscle overload, deconditioning, and systemic inflammation [31], which significantly impairs performance in essential daily activities requiring arm movements, including self-care and household tasks. Balance performance, evaluated through Balance Evaluation Systems Test, holds particular relevance as patients with COPD exhibit increased fall risk due to muscle weakness, altered body mechanics, oxygen desaturation, and medication side effects [32]. These balance impairments represent significant contributors to disability and reduced quality of life. The documented improvements in both upper limb strength and balance function therefore have substantial clinical implications, as they directly enhance patients’ functional independence, daily activity performance, and reduce fall risk [31]. These enhanced outcomes align with and extend existing evidence supporting both the effectiveness of motion-sensing game systems in improving postural control [33] and the superior benefits of comprehensive, whole-body engagement during PR [34]. The multidimensional improvements observed in our analysis suggest that MSG-based interventions provide a more holistic approach to addressing the complex functional limitations experienced by patients with COPD.

The significantly higher program completion rates represent a crucial advancement in addressing poor adherence, a persistent challenge in PR [35]. This is particularly significant as patients with COPD typically demonstrate notoriously low adherence rates due to symptom burden, comorbidities, and motivational challenges [36]. The direct relationship between improved adherence and enhanced rehabilitation outcomes highlights the clinical importance of these findings. Interventions improving adherence directly enhance rehabilitation outcomes. This enhanced engagement can be attributed to the gaming elements’ unique features: immediate feedback, achievement motivation, and progressive challenge levels [11]. The motion-controlled interaction promotes motor learning and functional movement patterns that directly translate to daily activities, addressing key limitations of traditional rehabilitation exercises [37]. These findings suggest that MSGs offer a promising approach to optimize exercise training outcomes while maintaining patient engagement.

#### Symptoms and Quality of Life

The significant reduction in dyspnea severity, particularly as measured by the modified MRC scale, indicates that PR+MSG offers enhanced symptom management benefits compared with conventional rehabilitation. The superior outcomes in modified MRC subgroups likely reflect this scale’s greater sensitivity in detecting functional dyspnea changes [38]. The moderate effect size and low heterogeneity across studies, despite varying gaming platforms and protocols, demonstrate consistent improvement in breathlessness management [39,40]. Dyspnea severity, while primarily a respiratory symptom, profoundly affects physical activity participation, psychological well-being, and social engagement, making its improvement clinically meaningful given its substantial impact on patients’ quality of life, daily activities, and health care utilization [3]. The enhanced symptom management can be attributed to the gaming environment’s distinctive features: progressive exercise intensity with engagement through distraction, real-time feedback for breathing control and pacing, and interactive elements that boost self-efficacy in managing breathlessness [41,42]. This aligns with previous research confirming that interactive gaming interventions effectively reduce perceived dyspnea through improved breathing control and exercise tolerance [3,43], suggesting a mechanistic pathway by which MSG intervention translates to functional improvement beyond conventional rehabilitation approaches.

Regarding HRQL, our findings demonstrate that PR+MSG yields significant improvements in patients’ perceived health status. This enhancement is particularly meaningful as HRQL represents a crucial patient-centered outcome that reflects the intervention’s comprehensive impact on daily functioning and overall well-being [44]. These results align with existing evidence suggesting that engaging, interactive exercise modalities enhance both physical outcomes and quality of life measures [45]. While moderate heterogeneity in the analysis reflects variations in implementation approaches and patient characteristics, the improved CAT scores suggest that game-based interventions effectively address multiple health status domains, including symptom burden, activity limitation, and psychosocial impact [46].

#### Psychological Outcomes

Our meta-analysis demonstrated significant improvements in psychological well-being with PR+MSG intervention, with notably greater effects on anxiety compared with depression scores. These findings align with previous evidence suggesting that interactive gaming environments can effectively mitigate psychological distress in chronic disease management [47,48]. The gaming elements’ distinctive features—achievement-oriented tasks, immediate feedback, and enhanced social interaction—appear particularly effective in reducing anxiety-related symptoms, which commonly impede exercise participation in patients with COPD [49-51]. This psychological benefit is especially relevant considering patients with COPD experience substantially higher rates of psychological distress compared with the general population, which directly contributes to disease progression, exacerbation risk, and mortality [52]. Additionally, reduced anxiety and depression levels contribute to better exercise adherence [53], while gamification elements help maintain long-term engagement through enhanced enjoyment and reduced perceived exertion [54]. These results support implementing comprehensive rehabilitation approaches that address both physical and psychological aspects of chronic respiratory diseases [3].

### Strengths and Limitations

This meta-analysis represents the first comprehensive review specifically focusing on MSG-assisted exercise training in patients with COPD, distinguishing itself from previous reviews that broadly examined various technological interventions. By systematically evaluating multiple outcomes (exercise capacity, symptoms, quality of life, and psychological parameters) through RCTs, this study provides the first synthesis of evidence on the comprehensive benefits of MSG-based rehabilitation. These findings offer practical guidance for enhancing patient care in contemporary clinical settings while suggesting promising directions for future research in technology-enhanced rehabilitation strategies.

Several key limitations warrant consideration in this study. First, methodologically, effective blinding was challenging due to the distinctive nature of game-assisted rehabilitation compared with conventional approaches, potentially introducing implementation bias. The heterogeneity among included studies manifested primarily in the varying gaming platforms and rehabilitation protocols, as well as inconsistent intervention specifications (intensity, frequency, and duration), which may have introduced clinical heterogeneity and affected outcome comparability. While we identified these possible contributing factors, the relatively small number of eligible studies precluded us from conducting meaningful subgroup analyses to quantify their specific contributions to the observed heterogeneity. Additionally, most included studies were constrained by small sample sizes and relatively short follow-up periods, limiting both the assessment of long-term efficacy and internal validity. The overall methodological quality of the included studies was suboptimal, with most evidence rated as “low” or “very low” quality. Furthermore, this study did not systematically evaluate the cost-effectiveness of implementing game-assisted rehabilitation systems in clinical settings. These limitations underscore the need for future research to conduct larger, well-designed RCTs with standardized intervention protocols, extended follow-up periods, and comprehensive cost-benefit analyses to provide more reliable theoretical guidance for clinical practice.

### Implications for Future Research and Practices

The findings demonstrate that incorporating MSGs into PR programs offers a promising strategy to enhance both clinical outcomes and patient engagement. This approach may be particularly beneficial for three key patient populations: those with poor adherence to conventional rehabilitation protocols, patients reporting exercise-related anxiety or low motivation, and individuals requiring concurrent physical and psychological support. Health care providers can leverage this technology-enhanced rehabilitation method to create more personalized and engaging treatment plans, potentially improving long-term adherence and rehabilitation outcomes. Additionally, the game-based format may help clinicians better monitor patient progress and adjust intervention intensity in real-time, though implementation should consider individual patient preferences, physical capabilities, and facility resources.

### Conclusion

This systematic review and meta-analysis suggests that PR+MSG may improve multiple clinical outcomes in patients with COPD, including exercise capacity, respiratory symptoms, HRQL, and psychological well-being. The observed heterogeneity in effectiveness across gaming platforms highlights the importance of personalized intervention selection based on individual patient characteristics and health care resource settings. While our results indicate potential benefits, the predominance of low-quality evidence underscores the need for risk-benefit assessment in clinical implementation of PR+MSG approaches. Future research priorities should include larger, methodologically rigorous RCTs, developing standardized intervention protocols, investigating sustained therapeutic effects, and conducting cost-effectiveness analyses to establish more definitive evidence for the optimal implementation of gaming technology in PR programs.

## Supplementary material

10.2196/69562 Multimedia Appendix 1 Comprehensive search strategy used to identify randomized controlled trials evaluating motion-sensing game–assisted pulmonary rehabilitation in patients with chronic obstructive pulmonary disease.

10.2196/69562 Multimedia Appendix 2 Risk of bias graph: review authors' judgements about each risk of bias item presented as percentages across all included studies.

10.2196/69562 Multimedia Appendix 3 Grading of Recommendations Assessment, Development, and Evaluations evidence profile and summary of findings for primary outcomes comparing motion-sensing game–assisted pulmonary rehabilitation versus routine pulmonary rehabilitation in patients with chronic obstructive pulmonary disease.

10.2196/69562 Checklist 1 Preferred Reporting Items for Systematic Reviews and Meta-Analyses (PRISMA) 2020 checklist.

## Abbreviations

6MWD: 6-Minute Walk Distance
CAT: COPD Assessment Test
COPD: chronic obstructive pulmonary disease
GRADE: Grading of Recommendations Assessment, Development, and Evaluations
HRQL: health-related quality of life
MD: mean difference
MRC: Medical Research Council
MSG: motion-sensing game
OR: odds ratio
PR: pulmonary rehabilitation
PR+MSG: motion-sensing game–assisted pulmonary rehabilitation
PRISMA: Preferred Reporting Items for Systematic Reviews and Meta-Analyses
PROSPERO: International Prospective Register of Systematic Reviews
RCT: randomized controlled trial
