# Supplementary material for: Efficacy of Motion-Sensing Game–Assisted Pulmonary Rehabilitation in Patients With Chronic Obstructive Pulmonary Disease: Systematic Review and Meta-Analysis of Randomized Controlled Trials
Source: JMIR Serious Games. 2025 May 29;13:e69562. doi: 10.2196/69562 (PMC12140370; doi:10.2196/69562)
Supplement: Multimedia Appendix 1 [file games-v13-e69562-s001.docx]

**Table S1. Comprehensive search strategy used to identify randomized controlled trials evaluating motion-sensing game-assisted pulmonary rehabilitation in patients with COPD.**

| **Search number** | **Query** | **Results** |
| --- | --- | --- |
| **PubMed** | | |
| 4 | ((((((((((("Randomized Controlled Trials as Topic"[Mesh]) OR ("Randomized Controlled Trial" [Publication Type])) OR ("Randomized Controlled Trial")) OR ("Randomized Controlled")) OR ("Randomized")) OR ("equally")) OR ("randomly")) OR ("RCT")) OR ("control group"))) AND ((((((("Pulmonary Disease, Chronic Obstructive"[Mesh]) OR ("chronic obstructive airway disease"[Title/Abstract])) OR ("COAD"[Title/Abstract])) OR ("chronic obstructive pulmonary disease"[Title/Abstract])) OR ("COPD"[Title/Abstract])) OR ("chronic airflow obstruction"[Title/Abstract])))) AND (((((((((((game*[Title/Abstract]) OR (gaming[Title/Abstract])) OR (exergame*[Title/Abstract])) OR (exergaming[Title/Abstract])) OR (gamification[Title/Abstract])) OR (virtual*[Title/Abstract])) OR (video*[Title/Abstract])) OR (interactive*[Title/Abstract])) OR (entertain*[Title/Abstract])) OR (computer[Title/Abstract])) OR ("Video Games"[Mesh])) | 341 |
| 3 | ((((((((("Randomized Controlled Trials as Topic"[Mesh]) OR ("Randomized Controlled Trial" [Publication Type])) OR ("Randomized Controlled Trial")) OR ("Randomized Controlled")) OR ("Randomized")) OR ("equally")) OR ("randomly")) OR ("RCT")) OR ("control group")) | 1,931,570 |
| 2 | (((((("Pulmonary Disease, Chronic Obstructive"[Mesh]) OR ("chronic obstructive airway disease"[Title/Abstract])) OR ("COAD"[Title/Abstract])) OR ("chronic obstructive pulmonary disease"[Title/Abstract])) OR ("COPD"[Title/Abstract])) OR ("chronic airflow obstruction"[Title/Abstract])) | 113,469 |
| 1 | ((((((((((game*[Title/Abstract]) OR (gaming[Title/Abstract])) OR (exergame*[Title/Abstract])) OR (exergaming[Title/Abstract])) OR (gamification[Title/Abstract])) OR (virtual*[Title/Abstract])) OR (video*[Title/Abstract])) OR (interactive*[Title/Abstract])) OR (entertain*[Title/Abstract])) OR (computer[Title/Abstract])) OR ("Video Games"[Mesh]) | 790,188 |
| **Web of Science** | | |
| 1 | TS=game* OR TS=gaming OR TS=exergame* OR TS=exergaming OR TS=gamification OR TS=virtual* OR TS=video* OR TS=interactive* OR TS=entertain* OR TS=computer | 7,075,322 |
| 2 | TS=“Pulmonary Disease, Chronic Obstructive” OR TS="chronic obstructive airway disease" OR TS="COAD" OR TS="chronic obstructive pulmonary disease" OR TS="COPD" OR TS="chronic airflow obstruction" | 129,600 |
| 3 | TS="Randomized Controlled Trial" OR TS="Randomized Controlled" OR TS="Randomized" OR TS="equally" OR TS="randomly" OR TS="RCT" OR TS="control group" | 2,331,864 |
| 4 | #1 AND #2 AND #3 | 324 |
| **Embase** | | |
| 4 | #1 AND #2 AND #3 | 519 |
| 3 | 'randomized controlled trial' OR 'randomized controlled' OR 'randomized' OR 'equally' OR 'randomly' OR 'rct' OR 'control group' | 2,718,994 |
| 2 | 'chronic obstructive lung disease':ti,ab,kw OR 'chronic obstructive airway disease':ti,ab,kw OR 'coad':ti,ab,kw OR 'chronic obstructive pulmonary disease':ti,ab,kw OR 'copd':ti,ab,kw OR 'chronic airflow obstruction':ti,ab,kw | 162,597 |
| 1 | game*:ti,ab,kw OR gaming:ti,ab,kw OR exergame*:ti,ab,kw OR exergaming:ti,ab,kw OR gamification:ti,ab,kw OR virtual*:ti,ab,kw OR video*:ti,ab,kw OR interactive*:ti,ab,kw OR entertain*:ti,ab,kw OR computer:ti,ab,kw | 1,000,315 |
| **CINAHL** | | |
| S1 | SU game* OR SU gaming OR SU exergame* OR SU exergaming OR SU gamification OR SU virtual* OR SU video* OR SU interactive* OR SU entertain* OR SU computer | 183,739 |
| S2 | SU "chronic obstructive airway disease" OR SU "COAD" OR SU "chronic obstructive pulmonary disease" OR SU "COPD" OR SU "chronic airflow obstruction" | 14 |
| S3 | AB "chronic obstructive airway disease" OR AB "COAD" OR AB "chronic obstructive pulmonary disease" OR AB "COPD" OR AB "chronic airflow obstruction" | 19,648 |
| S4 | SU "Randomized Controlled Trial" OR SU "Randomized Controlled" OR SU "Randomized" OR SU "equally" OR SU "randomly" OR SU "RCT" OR SU "control group" | 164,324 |
| S5 | AB game* OR AB gaming OR AB exergame* OR AB exergaming OR AB gamification OR AB virtual* OR AB video* OR AB interactive* OR AB entertain* OR AB computer | 149,312 |
| S6 | S2 OR S3 | 19,651 |
| S7 | S1 OR S5 | 286,844 |
| S8 | AB "Randomized Controlled Trial" OR AB "Randomized Controlled" OR AB "Randomized" OR AB "equally" OR AB "randomly" OR AB "RCT" OR AB "control group" | 408,158 |
| S9 | S4 OR S8 | 463,230 |
| S10 | S6 AND S7 AND S9 | 150 |
| **Cochrane Library** | | |
| 1 | (game*):ti,ab,kw OR (gaming):ti,ab,kw OR (exergame*):ti,ab,kw OR (exergaming):ti,ab,kw OR (gamification):ti,ab,kw (Word variations have been searched) | 11231 |
| 2 | (virtual*):ti,ab,kw OR (video*):ti,ab,kw OR (interactive*):ti,ab,kw OR (entertain*):ti,ab,kw OR (computer):ti,ab,kw (Word variations have been searched) | 135108 |
| 3 | #1 OR #2 | 140550 |
| 4 | ("chronic obstructive pulmonary disease"):ti,ab,kw OR ("chronic obstructive airway disease"):ti,ab,kw OR ("COPD"):ti,ab,kw OR ("COAD"):ti,ab,kw OR ("chronic airflow obstruction"):ti,ab,kw (Word variations have been searched) | 23312 |
| 5 | ("Randomized Controlled Trial"):ti,ab,kw OR ("Randomized Controlled"):ti,ab,kw OR ("Randomized"):ti,ab,kw OR ("equally"):ti,ab,kw OR ("randomly"):ti,ab,kw (Word variations have been searched) | 1330989 |
| 6 | ("RCT"):ti,ab,kw OR ("control group"):ti,ab,kw (Word variations have been searched) | 328096 |
| 7 | #5 OR #6 | 1424747 |
| 8 | #3 AND #4 AND #7 | 910 |
| **Scopus** | | |
| 1 | ( TITLE-ABS-KEY ( game* ) OR TITLE-ABS-KEY ( gaming ) OR TITLE-ABS-KEY ( exergame* ) OR TITLE-ABS-KEY ( exergaming ) OR TITLE-ABS-KEY ( gamification ) OR TITLE-ABS-KEY ( virtual* ) OR TITLE-ABS-KEY ( video* ) OR TITLE-ABS-KEY ( interactive* ) OR TITLE-ABS-KEY ( entertain* ) OR TITLE-ABS-KEY ( computer ) OR TITLE-ABS-KEY ( "video games" ) ) | 7,537,057 |
| 2 | ( TITLE-ABS-KEY ( "Pulmonary Disease, Chronic Obstructive" ) OR TITLE-ABS-KEY ( "chronic obstructive airway disease" ) OR TITLE-ABS-KEY ( "COAD" ) OR TITLE-ABS-KEY ( "chronic obstructive pulmonary disease" ) OR TITLE-ABS-KEY ( "COPD" ) OR TITLE-ABS-KEY ( "chronic airflow obstruction" ) ) | 117,336 |
| 3 | ( TITLE-ABS-KEY ( "Randomized Controlled Trial*" ) OR TITLE-ABS-KEY ( "Randomized Controlled" ) OR TITLE-ABS-KEY ( "Randomized" ) OR TITLE-ABS-KEY ( "equally" ) OR TITLE-ABS-KEY ( "randomly" ) OR TITLE-ABS-KEY ( "RCT" ) OR TITLE-ABS-KEY ( "control group" ) ) | 3,010,797 |
| 4 | #1 AND #2 AND #3 | 916 |
| **China National Knowledge Infrastructure** | | |
| 1 | （篇关摘：慢性阻塞性肺疾病 + 慢性阻塞性气道疾病 + COPD + COAD + 慢阻肺 + 慢性气道阻塞 + 慢性阻塞性肺病(精确)）AND（篇关摘：游戏 + 虚拟 + 娱乐 + 体感(精确)） | 77 |
| **Wanfang** | | |
| 1 | （主题:(慢性阻塞性肺疾病) or 主题:(慢性阻塞性气道疾病) or 主题:(慢阻肺) or 主题:(慢性气道阻塞) or 主题:(慢性阻塞性肺病) or 主题:(COPD) or 主题:(COAD) ） AND (主题:(游戏) or 主题:(虚拟) or 主题:(娱乐) or 主题:(体感)) | 137 |
