# Supplementary material for: Efficacy of Motion-Sensing Game–Assisted Pulmonary Rehabilitation in Patients With Chronic Obstructive Pulmonary Disease: Systematic Review and Meta-Analysis of Randomized Controlled Trials
Source: JMIR Serious Games. 2025 May 29;13:e69562. doi: 10.2196/69562 (PMC12140370; doi:10.2196/69562)
Supplement: Multimedia Appendix 2 [file games-v13-e69562-s002.docx]

A.


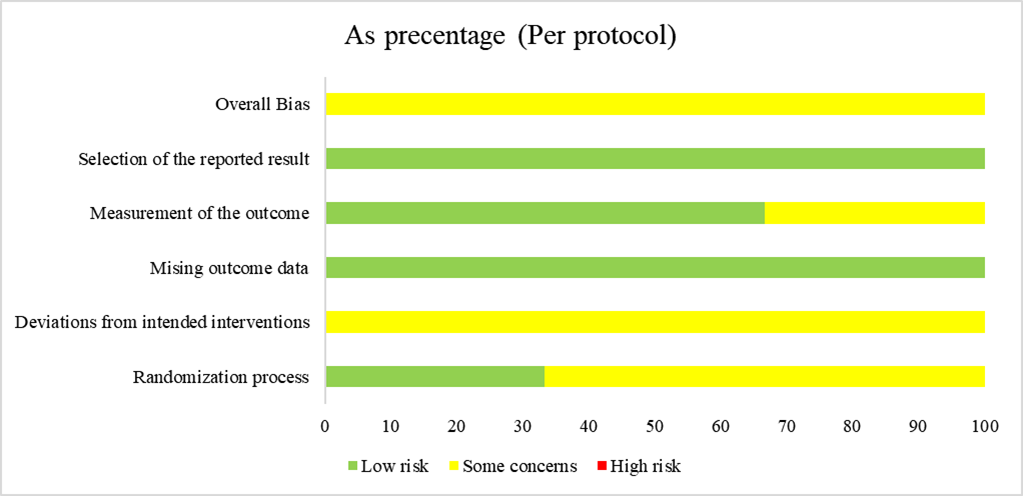


B.


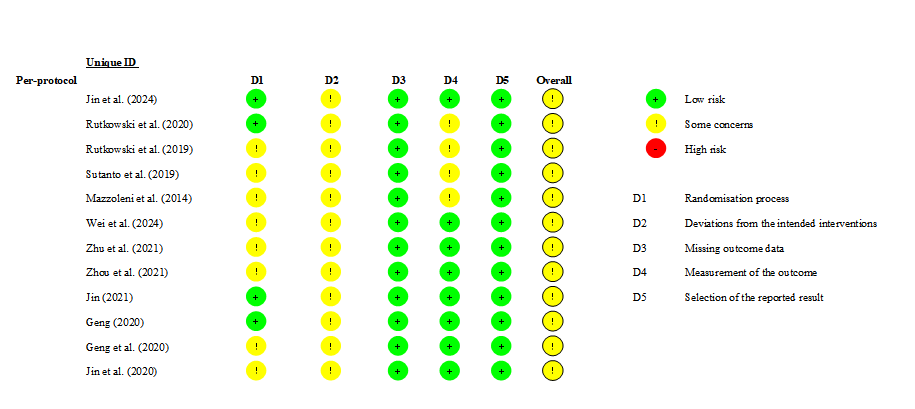


Figure S1. Risk of bias graph: review authors' judgements about each risk of bias item presented as percentages across all included studies (A). Risk of bias summary graph: review authors' judgements about each risk of bias item for each included study (B).
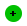
: low risk of bias;
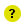
: some concerns;
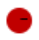
：high risk of bias.
