# Supplementary material for: Efficacy of Motion-Sensing Game–Assisted Pulmonary Rehabilitation in Patients With Chronic Obstructive Pulmonary Disease: Systematic Review and Meta-Analysis of Randomized Controlled Trials
Source: JMIR Serious Games. 2025 May 29;13:e69562. doi: 10.2196/69562 (PMC12140370; doi:10.2196/69562)
Supplement: Multimedia Appendix 3 [file games-v13-e69562-s003.docx]

**Table S2. GRADE evidence profile and summary of findings for primary outcomes comparing motion-sensing game-assisted pulmonary rehabilitation versus routine pulmonary rehabilitation in COPD patients.**

| **Quality assessment** | | | | | | | **No of patients** | | **Effect** | | **Quality ^a^** |
| --- | --- | --- | --- | --- | --- | --- | --- | --- | --- | --- | --- |
|  |  |  |  |  |  |  |  |  |  |  |  |
| **No of studies** | **Design** | **Risk of bias** | **Inconsistency** | **Indirectness** | **Imprecision** | **Other considerations** | **PR+MSG** | **PR** | **Relative**  **(95% CI)** | **Summary of**  **findings** |  |
| **6-Minute Walk Distance (6MWD)** | | | | | | | | | | | |
| 10 | 10 RCTs | serious^1^ | no serious inconsistency | no serious indirectness | serious^2^ | no serious inconsistency | 337 | 329 | MD 23.23 (14.47 to 31.99) | PR+MSG may improve walking distance. | ⊕⊕○○  LOW |
| **30-second Arm Curl Test (30sACT)** | | | | | | | | | | | |
| 4 | 4 RCTs | serious^1^ | serious^3^ | no serious indirectness | serious^2^ | reporting bias^4^ | 126 | 122 | MD 1.83 (0.63 to 3.03) | PR+MSG may improve upper limb muscle strength. | ⊕○○○  VERY LOW |
| **Brief Balance Evaluation Systems Test (Brief-BESTest)** | | | | | | | | | | | |
| 3 | 3 RCTs | serious^1^ | no serious inconsistency | no serious indirectness | serious^2^ | reporting bias^4^ | 130 | 132 | MD 2.34 (1.52 to 3.17) | PR+MSG may improve balance function. | ⊕○○○  VERY LOW |
| **Program Adherence** | | | | | | | | | | | |
| 2 | 2 RCTs | serious^1^ | no serious inconsistency | serious^5^ | serious^2^ | reporting bias^4^ | 83 | 79 | OR 3.00 (1.38 to 6.52) | PR+MSG may improve program adherence. | ⊕○○○  VERY LOW |
| **Severity of dyspnea** | | | | | | | | | | | |
| 6 | 6 RCTs | serious^1^ | no serious inconsistency | serious^5^ | serious^3^ | no serious inconsistency | 194 | 189 | MD -0.25 (-0.48 to -0.02) | PR+MSG may alleviate dyspnea. | ⊕○○○  VERY LOW |
| **Health-related quality of life (HRQL)** | | | | | | | | | | | |
| 2 | 2 RCTs | serious^1^ | serious^3^ | no serious indirectness | serious^3^ | reporting bias^4^ | 43 | 40 | MD -6.00 (-10.96 to -1.04) | PR+MSG may improve health-related quality of life. | ⊕○○○  VERY LOW |
| **Psychological State** | | | | | | | | | | | |
| 2 | 2 RCTs | serious^1^ | no serious inconsistency | no serious indirectness | serious^3^ | reporting bias^4^ | 56 | 53 | MD -2.41 (-3.42 to -1.39) | PR+MSG may alleviate anxiety. | ⊕○○○  VERY LOW |
| 2 | 2 RCTs | serious^1^ | no serious inconsistency | no serious indirectness | serious^3^ | reporting bias^4^ | 56 | 53 | MD -1.40 (-2.69 to -0.42) | PR+MSG may alleviate depression. | ⊕○○○  VERY LOW |

^a^ Symbols indicate the following strength of evidence:⊕⊕⊕⊕,High (We are very confident that the true effect lies close to that of the estimate of the effect.); ⊕⊕⊕○,Moderate (We are moderately confident in the effect estimate: The true effect is likely to be close to the estimate of the effect, but there is a possibility that it is substantially different.);⊕⊕○○, Low (Our confidence in the effect estimate is limited: The true effect may be substantially different from the estimate of the effect.); and ⊕○○○, Very low (We have very little confidence in the effect estimate: The true effect is likely to be substantially different from the estimate of effect.).

^1^ Most trials lack descriptions of randomisation methods and implementation of blinding.

^2^ The trials had a small total sample size or wide CIs.

^3^ High heterogeneity with I^2^ >70%.

^4^ Funnel plot is asymmetrical, or many trials included in the review do not contribute to the outcome.

^5^ The evaluation of the result is subjective.Different standards or methods in evaluating the result.

CI, confidence interval; MD, mean difference; OR, odds ratio; RCTs, randomised controlled trials.
